# *Mycobacterium tuberculosis *monoarthritis in a child

**DOI:** 10.1186/1546-0096-6-15

**Published:** 2008-09-18

**Authors:** Derek Rajakumar, Alan M Rosenberg

**Affiliations:** 1 Department of Pediatrics, University of Saskatchewan, Saskatoon, Canada

## Abstract

A child with isolated *Mycobacterium tuberculosis *monoarthritis, with features initially suggesting oligoarthritis subtype of juvenile idiopathic arthritis, is presented. This patient illustrates the need to consider the possibility of tuberculosis as the cause of oligoarthritis in high-risk pediatric populations even in the absence of a tuberculosis contact history and without evidence of overt pulmonary disease.

## Introduction

One-third of the world's population is infected with *Mycobacterium tuberculosis *and the global burden of tuberculosis continues to grow [1,2]. Approximately one-quarter to one-third of children with tuberculosis develop extrapulmonary involvement [3-5]. Skeletal tuberculosis, now rare since the advent of antituberculosis therapy, occurs in approximately 5% of children with extrapulmonary tuberculosis [3,4]. The vertebral body is the most common skeletal site involved followed by lower limb bones [5,6]. Rarely, intra-articular inflammation can occur in children either as a result of direct invasion of the tuberculous bacillus into the joint or as a consequence of an aseptic reactive arthritis (so-called Poncet's disease) related to an extra-articular tuberculous focus [3-20]. Intra-articular *Mycobacterium tuberculosis *infection is especially rare in children in the absence of associated pulmonary disease [7,8,13,14,17,19]. Consequently, delays in recognition and treatment of this diagnostically challenging condition occur.

We report a child with isolated *Mycobacterium tuberculosis *monoarthritis who presented with features initially suggesting oligoarthritis subtype of juvenile idiopathic arthritis (JIA). The patient we present illustrates the need to maintain heightened awareness that tuberculosis joint infection can occur in high-risk populations even in the absence of overt pulmonary involvement.

## Case report

At age 2 years 10 months this previously healthy North American Indian girl presented with a 3-week history of left knee swelling and morning stiffness without associated symptoms. There were no infectious contacts reported at first presentation. On initial physical examination, the left knee was moderately swollen and warm with signs of both intra-articular fluid and synovial hypertrophy. Flexion and extension were limited by 10 degrees. The child was afebrile and appeared otherwise healthy. There were no abnormal pulmonary signs and no peripheral lymphadenopathy. The remainder of the examination was normal.

Hemoglobin and white blood cell counts were normal. Platelet count (529 × 10^9^/L {normal 150–400 × 10^9^/L}) and C-reactive protein (10.6 mg/L {normal 0.0 – 7.0 mg/L}) were both elevated. The antinuclear antibody test was initially reported as negative (titer < 1:80) but on re-testing was weakly positive at a titre of 1:80. An annual tuberculin skin test, done 1 month prior to the onset of joint swelling, was negative.

Joint swelling persisted despite 3 weeks of non-steroidal anti-inflammatory therapy (naproxen, 20 mg/kg/day). The provisional diagnosis of oligoarthritis subtype of JIA was made and, because of persistent synovitis despite oral non-steroidal anti-inflammatory therapy, the left knee was injected with triamcinolone hexacetonide 1 mg/kg. Synovial fluid cell count revealed an absolute count of 14.4 × 10^9^/L of which 46% were neutrophils, 45% lymphocytes and 8% macrophages. Gram stain and routine bacterial cultures were negative. Synovial fluid glucose and protein levels were not measured.

There was prompt and complete resolution of the synovitis following intra-articular steroid injection but joint swelling recurred within 2 months and oral naproxen was resumed.

**Figure 1:**
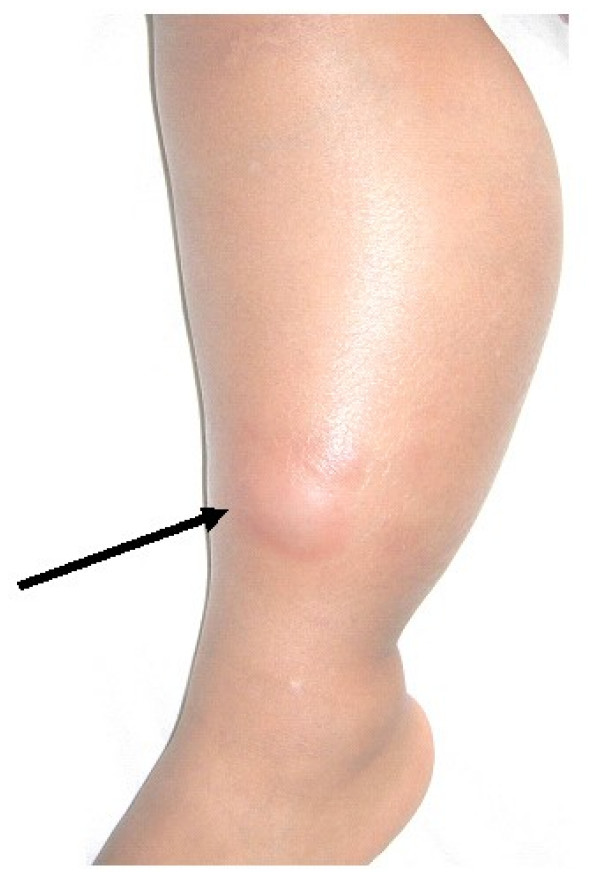
**Marked swelling of the left lower leg first developed 16 months after arthritis onset and 8 months following initiation of anti-tuberculosis treatment.** An erythematous bleb (arrow) was incised and the extruded fluid stained positive for acid fast bacilli.

Fourteen weeks after symptom onset and 8 weeks following intra-articular steroid injection the family became aware of an aunt who had been diagnosed with pulmonary tuberculosis 6 months following our patient's arthritis onset. Seven months after arthritis onset our patient had a normal chest radiograph. A repeat tuberculin skin test at that time was positive with 15 mm of induration; the patient had not received bacillus Calmette-Guerin vaccination. Eight months after arthritis onset a small left knee effusion was aspirated and culture was positive for *Mycobacterium tuberculosis*. Anti-tuberculous therapy (isoniazid and rifampin) was initiated. Joint swelling resolved within two months after initiation of treatment for tuberculosis and full joint range of motion was restored. Sixteen months after arthritis onset and 8 months following initiation of anti-tuberculous therapy the child developed diffuse circumferential swelling of the left lower leg that was not associated with pain or fever. The swelling was indurated, warm to the touch, and a 1 cm erythematous area was present overlying the distal medial aspect of the leg (Figure 1). No intra-articular fluid of the knee was detected. Incision and drainage of the erythematous patch yielded a milky discharge that stained positive for acid-fast bacilli; cultures of the discharge, however, were negative for *Mycobacterium tuberculosis *8 weeks after inoculation. Following approximately 8 weeks of drainage and 1 year of anti-tuberculous therapy the child remains well.

## Discussion

The patient we present illustrates the challenge in diagnosing isolated tuberculosis monoarthritis especially in the absence of a contact history and in the context of an initial negative tuberculin skin test and no overt pulmonary involvement. Our case also illustrates that the presentation of tuberculous arthritis can mimic oligoarthritis subtype of JIA. In our patient the diagnosis of tuberculous arthritis was not considered initially as there was no history of tuberculosis contact and a recent tuberculin skin test had been negative. Tuberculous arthritis was first considered when a contact with tuberculosis became evident six months after onset and was confirmed by skin test conversion to positive and eventual isolation of *Mycobacterium tuberculosis *from synovial fluid.

Tuberculous arthritis can result from direct invasion of *Mycobacterium tuberculosis *into a joint space (as in our case) or as a consequence of an aseptic reactive polyarthritis (Poncet's disease) [16,18]. Tuberculous arthritis associated with direct invasion is often monoarticular and characterized by an insidious onset [7,8,14,19] and not typically associated with signs of acute articular inflammation [13].

Early diagnosis of tuberculous arthritis is dependent on a high index of clinical suspicion that would be prompted particularly by knowing of an infectious contact or documenting conversion of a tuberculin skin test to positive. As illustrated by our patient, tuberculous arthritis can be difficult to distinguish from oligoarthrtis subtype of JIA in the context of an absent history of a tuberculosis contact and a recently documented negative skin test. In our case, tuberculous synovitis appeared to be a primary site of tuberculosis, rather than reactivation of a latent pulmonary or extra-pulmonary focus. Among 61 reports of tuberculous arthritis in children in which the knee was involved, including the present report, 32 (52.5%) had an apparent primary joint infection with no evidence of a pulmonary or other extra-articular focus [7,8,13,14,17,19]. Primary tuberculous arthritis in which one knee is the only joint involved, as in our case, has been reported rarely in children (Table 1) [7,8,13,14,19].

**Table 1:** Reported cases of tuberculous knee arthritis in children

**Author (reference)**	**Number of Cases**	**Number with joint/bone infection only**	**Appendicular joints involved**	**Presentation age**	**Diagnosis of tuberculosis**	**Chest radiograph**	**Skin test**
Southwood (18)	1	1	Knees, ankles	2 years	Positive culture from bone	Hilar adenopathy; no parenchymal involvement	Positive
Jacobs (14)	1	1	Knee	4 years	Histopathology (giant cells); cultures and stains for TB negative	Not stated	Initially negative then positive
Jacobs (14)	1	0	Knee	8 months	Positive synovial fluid culture	Positive	Initially negative then positive
Haygood (12)	45	Unknown	Hip – 18 Knee – 17 Ankle – 5 Shoulder – 2 Midfoot, elbow, wrist, hand – 1 each	6 months to 19 years	Positive culture, histopathology and/or guinea pig inoculation	Not stated	Not stated
Al-Matar (7)	1	1	Knee	2 years	Positive culture and histopathology	Normal	Positive
Al-Matar (7)	1	1	Knee	6 years	Positive culture and histopathology	Normal	Positive
Hoffman (13)	52	24	Knees	8 months to 13 years	Predominantly histopathologic but details not stated	Normal in 24 (47%)	91% Positive
Sawhney (17)	1	1	Knees, elbow, shoulder	13 years	Positive culture, polymerase chain reaction and histopathology	Normal	Positive
Aloui (8)	1	1	Knee	1 year	Histopathology of bone lesion	Normal	Negative
Aloui (8)	1	1	Knee	2 years	Histopathology and positive culture of bone lesion	Normal	Not stated
Uzel (19)	1	1	Knee	9 years	Synovial histopathology; cultures negative	Normal	Positive
Rajakumar (present case)	1	1	Knee	2 years	Synovial fluid culture	Normal	Initially negative then positive

Most patients presenting with tuberculous arthritis of a knee will present with indolent joint swelling due to synovial hypertrophy and fluid that can persist for years before diagnosis [13]. Approximately 15% of patients with tuberculous arthritis of a knee will have warmth and redness accompanying the swelling suggesting septic arthritis [13]. Non-specific indicators of inflammation, including fever and elevated acute phase reactants, are inconsistently present and cannot be relied on as discriminating diagnostic indicators. A positive Mantoux skin test is expected in almost all patients with tuberculous arthritis [13] but, as in our case, a positive skin test might not necessarily be present at initial arthritis presentation. Definitive diagnosis requires microbiological confirmation by isolating *Mycobacterium tuberculosis *from synovial fluid or synovium. Synovial membrane histopathology showing caseating granuloma, without documented microbiological confirmation, is suggestive of tuberculous arthritis. Polymerase chain reaction (PCR) has been reported to have low sensitivity (but high specificity) in detecting *Mycobacterium tuberculosis *from the knee in children [13]; however, the reliability of PCR in detecting *Mycobacterium tuberculosis *has been documented in other reports [21]. Synovial fluid analysis is not associated with sufficiently characteristic features in tuberculous synovitis to be consistently helpful diagnostically [22].

Tuberculous arthritis can be associated with erosion through the joint capsule to create draining sinuses, a circumstance which likely occurred in our patient and accounted for lower leg swelling and chronic drainage. Approximately 10% of patients with *Mycobacterium tuberculosis *knee arthritis will have a sinus at the time of initial presentation [13]. In our patient discharge from the sinus stained positive for acid-fast bacilli but cultures were negative suggesting the presence of non-viable, post-treatment remnants of the offending organism.

In high-risk populations with unexplained monoarthritis mimicking JIA, documenting a negative tuberculin skin test would seem prudent before proceeding to intra-articular corticosteroid injection. However, as demonstrated in our patient in whom a negative skin test was recorded 1 month prior to the onset of joint swelling, a delay in conversion to a positive skin test can occur after exposure to *Mycobacterium tuberculosis*. In our patient, treatment with intra-articular corticosteroids resulted in prompt but unsustained resolution of synovitis. The recurrence of joint swelling within 2 months of injection might have been an indication that the synovitis was different than oligoarthritis subtype of JIA in which a more sustained response to intra-articular triamcinolone hexacetonide therapy is typical.

In our patient intra-articular corticosteroids, which traditionally have been considered to be contraindicated in septic arthritis, were administered inadvertently but without any apparent adverse consequences. There are no substantive data to support the use of intra-articular steroids as adjunctive therapy in septic arthritis. However, concomitant use of systemic antibiotics and intra-articular steroids are not deleterious in experimental animals [23] and it has been suggested that the use of intra-articular steroids might be chondroprotective [24]. Thus, it is conceivable, though unproven, that intra-articular corticosteroids could be effective adjunctive therapy in septic arthritis by blunting inflammatory mediators that contribute to cartilage and bone degradation. The theoretical role for intra-articular steroids as adjunctive therapy for septic arthritis might prove to be analogous to the putative therapeutic efficacy of steroids in sepsis occurring at other sites including in the treatment of tuberculosis of the pericardium, pleura and meninges [25].

## Conclusion

Our experience illustrates the need to maintain a high index of suspicion for the possibility of tuberculosis as the cause of arthritis in high-risk populations even when a tuberculosis contact is not immediately evident, when there is no overt pulmonary involvement, and when a recent tuberculin skin test is negative.

## Competing interests

The authors declare that they have no competing interests.

## Authors' contributions

Both authors read and approved the final manuscript. DR reviewed and summarized the patient's medical history, undertook a literature review pertinent to the condition and contributed to the preparation of the manuscript. AR provided medical care for the child, undertook the investigations that led to the diagnosis, identified the subject as notable and worthy of reporting, and assisted in the literature review and preparation of the manuscript.
